#  A questão das urgências: uma porta para os direitos humanos em saúde

**DOI:** 10.1590/0102-311XPT168123

**Published:** 2023-12-04

**Authors:** José Ricardo de Carvalho Mesquita Ayres

**Affiliations:** 1 Faculdade de Medicina, Universidade de São Paulo, São Paulo, Brasil.

Uma verdadeira aula sobre o Sistema Único de Saúde (SUS)! Na verdade, mais do que isso, a leitura de *Acesso às Urgências e Atenção Hospitalar: Uma Questão de Direitos Humanos*^1^, escrito por Gisele O’Dwyer & Mariana Konder, nos convida a visitar, ou revisitar, com informações objetivas, claras e atualizadas não apenas a estrutura e operação do SUS brasileiro, mas também aquelas de diversos outros países, ainda que na forma de cotejamentos. Isso, é claro, tendo como foco central o lugar estratégico (e problemático) que o atendimento às urgências ocupa nesses diversos contextos. Mas o que é extremamente interessante é o fato de que essas incursões ao contexto do SUS e de outras experiências internacionais não cumprem um papel de mera demonstração enciclopédica ou de resposta a uma exigência protocolar de natureza acadêmica. Elas estão no texto na justa medida e necessidade em que essa contextualização e cotejamento permitem às autoras compartilhar seus pontos de vista, sua aproximação crítica às urgências no SUS, desde a assumida perspectiva de quem se coloca decidida e entusiasticamente em defesa do SUS, parafraseando Latour.

É sob essa perspectiva que as autoras chamam a atenção para um aspecto fundamental, e que muitas vezes nos passa despercebido: o acesso às urgências é, simultaneamente, um analisador do estado de saúde do sistema de saúde e um potente recurso para a promoção da saúde desse sistema - e, por conseguinte, dos cidadãos que dele dependem para promover, proteger e recuperar a sua própria. Elas nos mostram como a diferenciação e articulação entre urgências e emergências e as respectivas condições estruturais e operacionais de seus processos de trabalho configuram um ponto crítico para o bom funcionamento do SUS. Da mesma forma, e em relação com essa delicada e estratégica diferenciação/articulação, mostram-nos o papel a ser desempenhado, de um lado, por uma rede hospitalar bem dimensionada e preparada e, de outro lado, por uma rede de atenção primária à saúde (APS) igualmente bem planejada e capacitada.

As autoras deixam claro como uma rede hospitalar funcional é essencial para garantir um fluxo adequado entre urgências, emergências, acesso a intervenções que envolvem tecnologias materiais que dependem do complexo hospitalar e regresso ao plano ambulatorial, preferencialmente para o seguimento longitudinal da APS. O capítulo 3 nos mostra, de modo fundamentado em dados, como problemas de diversas ordens têm tornado os hospitais em barreiras para o bom funcionamento das urgências e emergências, porque represam as emergências com pouca disponibilização do acesso ao cuidado hospitalar, com todos os efeitos de retroação sobre as instâncias que o antecedem na linha de cuidado: emergências lotadas, queda na qualidade do cuidado oferecido e sobrecarga dos profissionais - e eu acrescentaria a importância desse desarranjo sobre a própria segurança dos pacientes ^2^ -, urgências também sobrecarregadas com demandas que fogem à sua competência, unidades básicas sem alternativas para responder adequadamente às situações de urgência e emergência, causando prejuízos para o cuidado e comprometimento de sua legitimação social.

Por sua vez, se é verdade que uma APS com cobertura adequada tende a dar maior visibilidade e acesso a situações que requerem atendimentos de urgência e emergência e, nesse sentido, a aumentar potencialmente a demanda por esses atendimentos, é verdade, também, que um cuidado longitudinal de qualidade, com ações consistentes de promoção da saúde, prevenção, diagnóstico precoce e tratamento adequado, e mesmo de atendimento a algumas modalidades de urgência, tenderá a tornar mais seletiva e adequada a demanda às emergências e aos hospitais. Assim, embora a APS sozinha certamente não consiga responder à crescente complexidade das demandas atuais por saúde ^3^, não há dúvida de que ela é estratégica para fazer o conjunto do sistema de saúde funcionar bem, e o efeito positivo disso é percebido especialmente nos indicadores de saúde dos grupos populacionais em situação de maior vulnerabilidade em saúde ^4^^,^^5^. Além disso, a incapacidade de absorção das urgências por uma APS fragilizada, transferindo a demanda para serviços de emergência 24 horas, em geral hospitalares, sobrecarregados e despreparados para a complexidade de suas demandas, tornam esse grupos - idosos, portadores de doenças, urgências sociais, doenças psíquicas e sofrimento mental, pessoas com deficiência, pessoas em conflito com a lei, urgências ginecológicas, mulheres vítimas de violência, pacientes terminais, população negra e indígena e populações LGBTQIA+ - mais suscetíveis a situações de violência institucional.

Este talvez seja o traço mais original e fecundo da obra: o ato de situar a questão do acesso às urgências na dinâmica de suas relações com as diferentes instâncias e níveis que compõem um sistema de saúde, sem descuidar das especificidades técnicas desse componente (capítulo 2). Estas últimas são descritas e discutidas com minúcia e rigor, mas sem perder em clareza e didatismo. Assim situada, a tecnicidade se vê livre de uma leitura tecnicista das urgências, emergências e atenção hospitalar. Elas são parte de redes (aliás, o conceito de rede permeia todo o texto, desde a útil introdução no capítulo 1) livres ou não das injunções históricas e políticas, de que nos dão conta as análises comparativas das urgências em sistemas de saúde universais (capítulo 4), assim como a discussão emblemática da recente experiência brasileira com a pandemia de COVID-19.

Mas há ainda outro elemento de fundamental importância que desejo destacar. Não seria possível o sucesso alcançado por esse texto na leitura dinâmica e contextual de um tema tão atravessado por normatividades técnicas, não seria possível evitar tecnicismos na abordagem de um aspecto tão instrumental da organização das práticas de saúde se não houvesse uma referência, uma “totalidade compreensiva” a ancorar as análises em um forte “compromisso prático”, no sentido ético e político, com a equidade e com as populações vulnerabilizadas ^6^. E essa referência, que aparece no subtítulo do livro, mas só é tratada abertamente em seu final, são os direitos humanos.

Com efeito, é essa referência que confere ao texto esse frescor do vivido, essa concretude que permite resistir a leituras das urgências e da assistência hospitalar como um sistema abstrato de relações mecânicas, regidas por teleologias de caráter estritamente instrumental. É essa referência que possibilita a crítica imanente da técnica, por dentro da técnica, mas não reduzida à tecnicalidade. É ela que, como já dito, permite às autoras criticar o SUS para defendê-lo. Está aqui uma inconfundível marca da afiliação à tradição da Saúde Coletiva. Só ela permite entender que o último parágrafo de um texto sobre acesso a urgências e assistência hospitalar convoque seus leitores a “*lutar contra as insuficiências estruturais e contra a violação dos direitos humanos, traduzidas na não concretização da integralidade, da universalidade e da equidade nos serviços públicos de saúde*” (p. 149).

Por isso, vou me permitir, como resenhista, fazer uma ousada proposta aos leitores, ainda que correndo o risco de contrariar suas autoras: que comecem a leitura do livro do fim para o começo. Talvez essa pequena “subversão” ajude a fazer jus à potente hermenêutica subjacente ao excelente e inspirador trabalho de Gisele e Mariana.
